# Ultrastructural Changes of Airway in Murine Models of Allergy and Diet-Induced Metabolic Syndrome

**DOI:** 10.1155/2013/261297

**Published:** 2013-09-10

**Authors:** Geeta Devi Leishangthem, Ulaganathan Mabalirajan, Vijay Pal Singh, Anurag Agrawal, Balaram Ghosh, Amit Kumar Dinda

**Affiliations:** ^1^Renal Pathology Laboratory, Department of Pathology, All India Institute of Medical Sciences, New Delhi 110029, India; ^2^Molecular Immunogenetics Laboratory and Center for Translational Research in Asthma and Lung Disease, CSIR-Institute of Genomics and Integrative Biology, Delhi 110007, India

## Abstract

Studying ultrastructural changes could reveal novel pathophysiology of obese-asthmatic condition as existing concepts in asthma pathogenesis are based on the histological changes of the diseased airway. While asthma is defined in functional terms, the potential of electron microscopy (EM) in providing cellular and subcellular detail is underutilized. With this view, we have performed transmission EM in the lungs from allergic mice that show key features of asthma and high-fat- or high-fructose-fed mice that mimicked metabolic syndrome to illustrate the ultrastructural changes. The primary focus was epithelial injury and metaplasia, which are cardinal features of asthma and initiate airway remodeling. EM findings of the allergically inflamed mouse lungs correlate with known features of human asthma such as increased mitochondria in airway smooth muscle, platelet activation and subepithelial myofibroblasts. Interestingly, we found a clear and unambiguous evidence to suggest that ciliated cells can become goblet cells using immunoelectron microscopy. Additionally, we show for the first time the stressed mitochondria in the bronchial epithelia of high-fat- or high-fructose-fed mice even without allergen exposure. These results may stimulate interest in using EM in understanding novel pathological mechanisms for different subtypes of asthma including obese asthma.

## 1. Introduction

**Figure 1:**
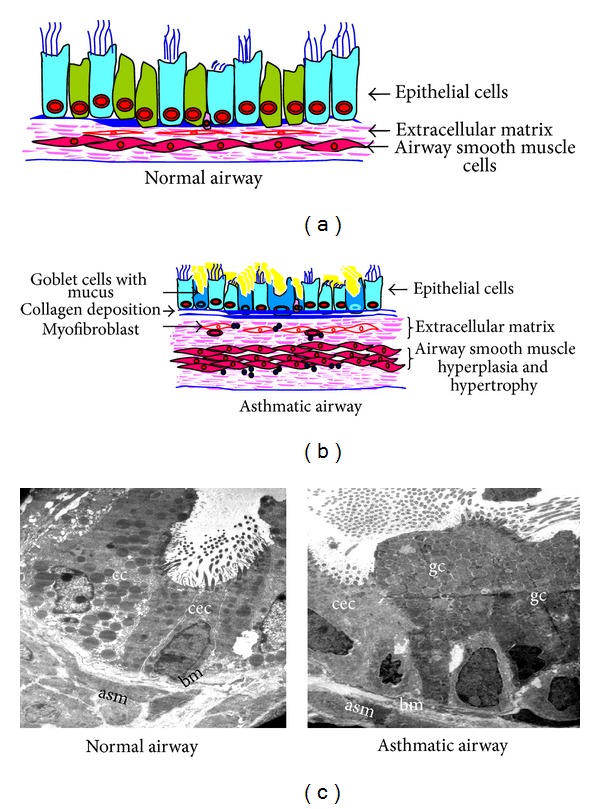
Components of structural changes of asthmatic airway. (a) and (b) Schematic diagrams show major components of normal and asthmatic airways. (c) Transmission electron microscopy (TEM) of lung to show the components of airway remodeling. Normal airway shows almost equal portions of ciliated epithelial cells (cec) and Clara cells (cc) and occasional basal cells and no mucous cell, thin basement membrane (bm), attenuated fibroblast sheath, and thin layer of airway smooth muscle (asm). Asthmatic airway shows the predominance of goblet cells (gc), thick basement membrane (bm), thickened (myo) fibroblast sheath, and hypertrophy and hyperplasia of airway smooth muscle (asm). Images are at 880X magnification.

Asthma, a chronic airway disease, is characterized by reversible airflow obstruction, airway inflammation, airway hyperresponsiveness (AHR), and structural changes referred to as airway remodeling [1, 2]. Asthma can be described in clinical, physiological, immunological, and pathological terms, each providing its own unique context in understanding asthma pathogenesis. Though this description seems to be simple, various endotypes/phenotypes of asthma have been recently demonstrated indicating the complexity of asthma [3–5]. Similar complexity also exists in the responsiveness to available antiasthma medications. The difficult-to-treat or severe or refractory asthmatics are responsible for significant health and economic burden of asthma even though they are just 10% of all asthmatics [6–9]. In this context, it has been demonstrated that asthma severity correlates very well with increased body mass index [10, 11]. As obese-asthmatic condition does not fall in the description of general asthma, there is necessity to understand the novel mechanisms for this condition. Muscular disease, inflammation dominant disease, airway remodeling, and epithelial injury are the historical descriptions or concepts of the asthma [12–16]. Indeed, current evidence suggests that airway inflammation is only one facet of the disease. Indeed, there is no satisfactory correlation between airway inflammation and airway hyperresponsiveness [17], although there is a good correlation between structural changes of the airway and AHR [18]. Repeated allergen exposures lead to numerous cycles of inflammation and healing and alter the structure of airway called airway remodeling (Figure 1(b)). This involves almost every part of airway from the epithelium to the adventitia. Epithelial hypertrophy and hyperplasia, goblet cell metaplasia, subepithelial fibrosis, basement membrane thickening, and hypertrophy and hyperplasia of airway smooth muscle are major features of airway remodeling in asthmatic airway compared to normal airway (Figures 1(a) and 1(b)) [2]. Each of these changes has the potential to alter airway physiology to promote airway narrowing and hyperresponsiveness [19, 20]. The structural pathological findings of asthma not only provide insights into its pathophysiology but also provide a relatively holistic view. Bronchial epithelium is now considered as central in asthma pathogenesis [2], and epithelial injury is a crucial phenomenon to initiate the airway remodeling by activating epithelial mesenchymal trophic unit (EMTU). Currently epithelial injury has been considered as a central feature in asthma pathogenesis. Evidently, it has been demonstrated that cytokines secreted by stressed airway epithelia can decide the immune status of lung [21–23]. It is being believed that exploring airway epithelia could explain the various lacunae in asthma pathogenesis such as the complexity. Thus, the concepts for asthma pathogenesis have been changed from a defect of smooth muscle to airway remodeling to epithelial injury [24]. Interestingly, all these concepts are based on the histopathological observations. These indicate that novel pathophysiology can be revealed through miniscule examination of the diseased airway. This will require precise definition of its features, at the tissue, cellular and even subcellular level. While the first is readily demonstrable through light microscopy, we consider that transmission electron microscopy will be an invaluable tool in precise delineation of the minutiae such as alteration in subcellular organelles, type of epithelial cells, and type of secretory granules present in epithelia. (Figure 1(c)). We have hypothesized that obese-asthmatics and chronic asthmatic condition may be explored by TEM to get miniscule information that may uncover novel pathophysiological mechanisms. TEM is an essential tool for examining ultrastructural details compared to other methods, even confocal microscopy [25]. Although there are existing reports that describe specific structural and minute changes of asthmatic lung both in mice and human, we did not find any suitable collection or review of TEM structural changes in mouse models of asthma. We consider this to be an important gap that may be hindering routine application of EM technology towards the understanding of asthma, and we have put together a collection of representative images from our lab illustrating the important changes in lung structure in standard acute and chronic mouse models of asthma processed for transmission electron microscopy (TEM) and immunoelectron microscopy. In this study, we have also performed TEM study in lungs of mice which were fed either high-fat- or high-fructose diet to see the ultrastructural changes in bronchial epithelia.

## 2. Materials and Methods

### 2.1. Mouse Model of Allergic Airway Inflammation

The 6–8 weeks male Balb/c mice (Central Drug Research Institute, Lucknow, India, or National Institute of Nutrition, Hyderabad, India) were obtained and maintained in CSIR-Institute of Genomics and Integrative Biology, Delhi, India. All the animal protocols were approved by the Institutional Animal Ethics Committee. There were two experimental groups in a murine model of allergic airway inflammation: SHAM (normal mice), and OVA (asthmatic mice) and each group contains at least 6 mice. Each mouse was sensitized and challenged as described earlier [26–31]. Briefly, each mouse was sensitized by three injections (intraperitoneal) of 50 *μ*g OVA (grade V chicken egg ovalbumin, Sigma, St. Louis, MO, USA) in 2 mg alum (OVA group) or 2 mg alum alone (SHAM group) on days 0, 7, and 14. After a week, these mice were challenged with 3% OVA (OVA group) or PBS (SHAM group) 30 min a day for seven consecutive days in acute model and 30 min a day in alternative days for 4 weeks in chronic model [32]. It is to be noted that we have not described the asthma symptoms such as airway hyperresponsiveness and immunological alterations such as IgE and cytokines and light microscopy findings such as airway inflammation, goblet cell metaplasia, and subepithelial fibrosis in these models as these are all the well-established models in our lab [26–32]. 

 To estimate the ultrastructural changes in lungs, the combined in situ perfusion and immersion fixation was performed as described previously [33]. Briefly, midsternal splitting incision was performed in deeply anaesthetized mice (sodium pentobarbitone) to open the thoracic cavity. The 18 gauze plastic cannula was inserted into the tip of the heart. Complete exsanguination was done using PBS that was located one meter above the mouse carcass followed by switching over to the cacodylate buffer. This fixation was performed at room temperature for at least 30 min with a flow rate of approximately 3 mL/min. The fixation was stopped when the organs became hard. Fixed lungs were again immersed into the fixative containing 2.5% glutaraldehyde. After overnight fixation, fixed lungs were dissected with Dissection microscope (SZX-12, Olympus) to find the first generation bronchi. Dissected bronchi were further processed to make blocks which were further stained and visualized with transmission electron microscope (Morgagni 268D, Fei Electron Optics). Immunoelectron microscopy was performed as described earlier [34]. Briefly, ultrathin sections (60 nm) of lungs were made and mounted on nickel grid; resin was removed with 2% sodium metaperiodate, blocked in 2% skimmed milk, and incubated with Muc 5ac, a primary antibody (in a moist chamber for overnight at 4°C) and further with secondary antibody (TAAB immunogold conjugate EM protein A, GEM020, A: 15 nm,) followed by counterstaining with uranyl acetate and lead citrate, and stained sections were viewed under electron microscope (CM10 Philips/*Morgagni* 268D, Fei *Electron* Optics). In negative control experiments, primary antibody was omitted. 

### 2.2. High-Fat-Fed Mouse Model

The 4-5 weeks male C57BL/6 mice (National Institute of Nutrition, Hyderabad, India) were obtained and maintained in CSIR-Institute of Genomics and Integrative Biology, Delhi, India. The animal protocols were approved by the Institutional Animal Ethics Committee. There were three experimental groups in this model: control mice (normal mice which were given standard rodent chow diet), high-fat mice (mice which were given high-fat diet in which fat alone can provide 60% of energy), and high-fructose mice (mice which were given high-fructose diet in which fructose alone can provide 70% energy), and each group contains at least 6 mice. These special diets were obtained from Research diet Inc., USA. These mice were kept in individual ventilated cages for twenty weeks, and fresh food and purified water were provided ad libitum on daily basis with proper recording of food intake and weight. At 19th week after housing, body mass, BP, and airway hyperresponsiveness to methacholine were measured. At 20th week, mice were sacrificed, and in situ perfusion and fixation was performed as described above to make lung blocks which were further stained and visualized with transmission electron microscope. It is to be noted high-fat- or high-fructose-fed mice had developed the features of metabolic syndrome such as hyperglycemia, hyperinsulinemia, and increased blood pressure. Only high-fat rather than high-fructose-fed mice gained double the initial weight.

## 3. Results and Discussion

### 3.1. Eosinophil Activity and Platelet Sequestration

**Figure 2:**
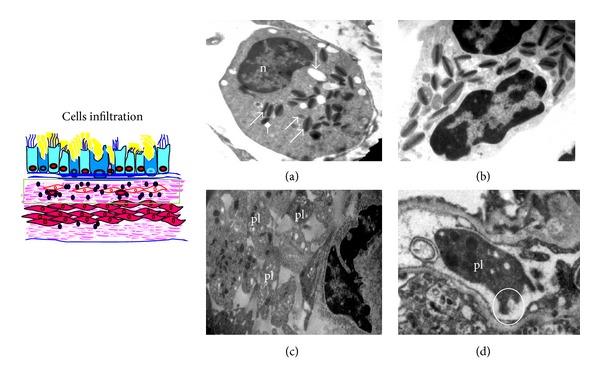
Eosinophil activity and platelet sequestration in asthmatic airway. TEM of eosinophil shows the peculiar granules with electron dense core surrounded by electron lucent matrix. Asthmatic mice of subacute (a) model show more granules (white arrows) with loss of either core or matrix indicating more degranulation and thus more activation, and in contrast asthmatic mice of chronic (b) model show more intact granules indicating less degranulation and thus less activation. (c) Numerous platelets (pl) are seen in and around the blood vessel in asthmatic mice, and higher magnification (d) shows the process of platelet sequestration (circle), n, nucleus. Images are at 3200X magnification.

We have developed allergen induced murine asthma model by OVA-sensitization and challenge. These mice developed methacholine-induced airway hyperresponsiveness, infiltration of various inflammatory cells including eosinophils both in the perivascular and peribronchial regions of bronchi, increase in the OVA-specific IgE levels, and increased goblet cell metaplasia compared to normal control mice [26–33]. As this paper demonstrates the ultrastructural changes, the above findings are not mentioned here. While airway inflammation is best quantified by light microscopy, important insights about alteration in cell function can be gleaned from EM data. As shown in Figures 2(a) and 2(b), eosinophils have peculiar granules which have two parts: inner core and outer matrix. These two parts are preserved in inactive or intact granules, and loss of core and/or matrix indicates active granules. Though the existence of eosinophil degranulation in experimental asthma is controversial [35], we found eosinophils with many partial active granules in the lung of subacute model (Figure 2(a)) but mostly inactive granules in chronic model (Figure 2(b)). This corresponds to higher degree of inflammatory changes in the former, despite similarly elevated levels of IL-5, an eosinophil survival factor in either model. Interestingly, we observed numerous platelets in and around the blood vessels of asthmatic lung compared to very few or none in normal mice (Figure 2(c)). Additionally, signs of sequestration indicated the activation of platelets (Figure 2(d)). It is well known that platelets participate in airway remodeling through release of granule contents such as platelet derived growth factor (PDGF). EM may be suitable for qualitative or quantitative assessment of platelet aggregation as well as activation in asthma. Allergen induces the migration of platelets to lung tissue in allergic asthma [36]. 

### 3.2. Mucous Metaplasia and Epithelial Injury

**Figure 3:**
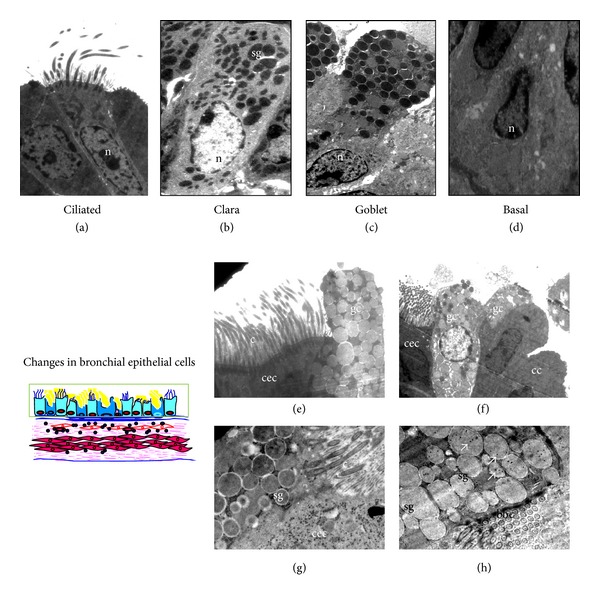
Different types of major epithelial cells present in the airway and Goblet cell metaplasia. (a) Ciliated: columnar shaped with elongated nuclei (n) near the base, and electron lucent cytoplasm. (b) Clara: dome-shaped with circular electron dense secretory granules (sg). (c) Goblet: mucus containing membrane bound electron lucent vacuoles in cytoplasm and (d) Basal cell: triangle shaped cell located just above the basement membrane with small basal nuclei (n). (e) TEM of the bronchial epithelia of asthmatic mice showed an abundance of goblet cells (gc) with numerous secretory granules (sg) containing mucus, c, cilia. (f) Clara (cc) and (g) Ciliated epithelial cells (cec) also show secretory granules. (h) Immunogold labeling with Muc5ac show the positive localization of Muc5ac as shown by the presence of gold particles (15 nm) in secretory granules (sg) of ciliated epithelia of asthmatic mice (bbc—basal bodies condensation). Images are at 880X (a)–(f) 3200X (g)-(h) magnifications.

Airway epithelial cells are the major barrier between the environment and internal milieu of the lung. There are four major types (ciliated, Clara, mucous/goblet, and basal, Figures 3(a)–3(d)) [37]. The proportion and location of each cell type vary depending on the species [38]. In normal mice, Clara cells comprise 52% of all epithelial cells in the trachea and approximately 33% in terminal bronchioles. In contrast, Clara cells are almost absent in proximal airways of humans, being restricted to the distal small airways [38]. Ciliated cells can be recognized by the characteristic cilia and ciliary bars (Figure 3(a)). Ciliated cells are the prominent cell type in human bronchi, while both Clara and ciliated cells are prominent in mice [37]. Clara cells can easily be distinguished by their characteristic electron-dense granules containing the Clara cell secretory protein (CCSP) (Figure 3(b)). Goblet cells are packed with electron-lucent vesicles that contain mucin (Figures 3(c) and 3(e)), and triangle-shaped basal cells are located just above the basement membrane with small basal nuclei (Figure 3(d)). 

Goblet cell metaplasia is considered to be a very important pathological event in asthma since mucus hypersecretion and plugging of airways lead to airway obstruction, airway hyperresponsiveness [39], and even fatal asthma [1]. Similar to human asthma, mouse models of asthma are associated with increased numbers of goblet cells, predominantly in the proximal airways. It was initially suggested that these increased numbers of goblet cells are either due to metaplasia of ciliated cells and/or proliferation of existing goblet cells [40]. However, later investigations found that these cells result from increased mucin expression in Clara cells [41, 42]. Careful analysis of goblet cells in our EM data suggests that both views are correct. We commonly see cells with both electron-dense and electron lucent secretory granules conforming to the current paradigm that Clara cells additionally develop mucin vesicles (Figure 3(f)). However, we also found secretory granules in ciliated epithelia, indicating the possible role of ciliated cells in mucus hypersecretion (Figure 3(g)). Immuno-EM confirmed that Muc5Ac localized to these granules (Figure 3(h)). Note the origin of the cilia from the cell in Figure 3(g) and the ciliary bars in Figure 3(h) confirming that these are ciliated cells. This is an important finding relevant to human asthma where the major proportion of epithelia of proximal airways is ciliated cells and merits further investigation.

**Figure 4:**
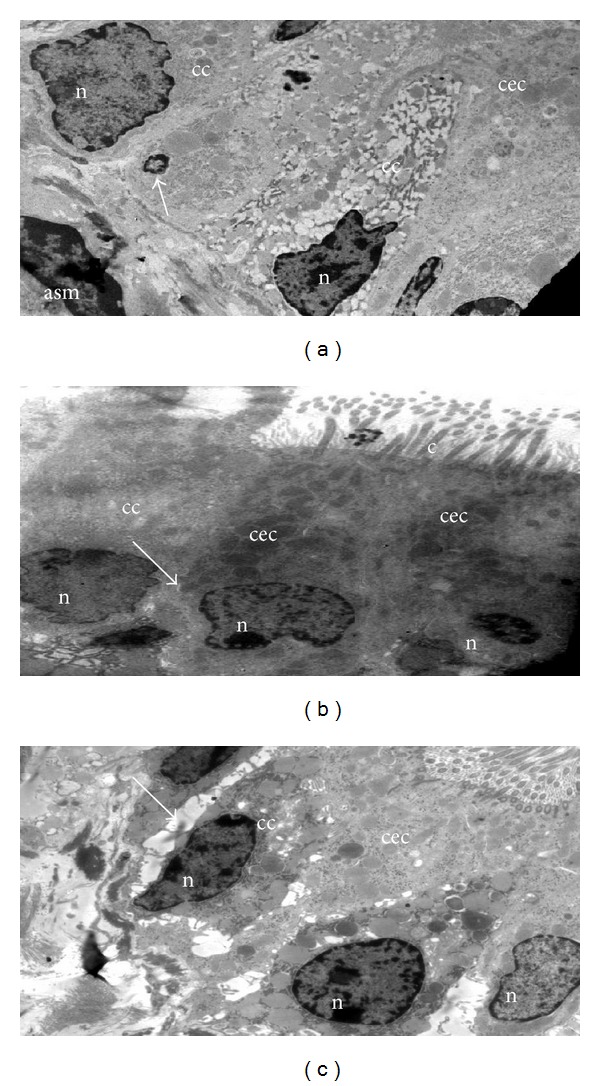
Epithelial injury. (a) Asthmatic mice show the shrinkage of bronchial epithelia (arrow) with pyknotic nucleus and marginated heterochromatin. Normal airways (b) show thin and regular intercellular space as compared to that of asthmatic airways. (c) Widening of intercellular space; cc, clara cells; asm, airway smooth muscle; cec, ciliated epithelial cells; n, nucleus; c, cilia. Arrows in (b) and (c) indicate the intercellular space between adjacent bronchial epithelia. Images are at 880X magnification.

Some other observations seem worthy of mention. Available evidence clearly indicates that there are more shedding epithelial cells in asthmatic patients [43]. It has been demonstrated that ciliated epithelia were found in BAL and sputum of asthmatic patients, and ciliated epithelia in BAL fluid positively correlated with severity of the disease including airway hyperresponsiveness [43]. It has been shown that there is increased caspase 9 activity and DNA fragmentation in asthmatic bronchial epithelia [44], both in human and mice. As shown in Figure 4(a), epithelial cells from allergically inflamed lungs show changes characteristic of apoptosis. Normally, adjacent bronchial epithelia are interconnected by tight junctions resulting in a thin and regular intercellular space (Figure 4(b)). In mice with experimental asthma, there is a widening of these spaces (Figure 4(c)). This widening might be because of reduction in tight connections [45]. 

### 3.3. Mitochondrial Structural Changes in High-Fat- or High-Fructose-Fed Mice

**Figure 5:**
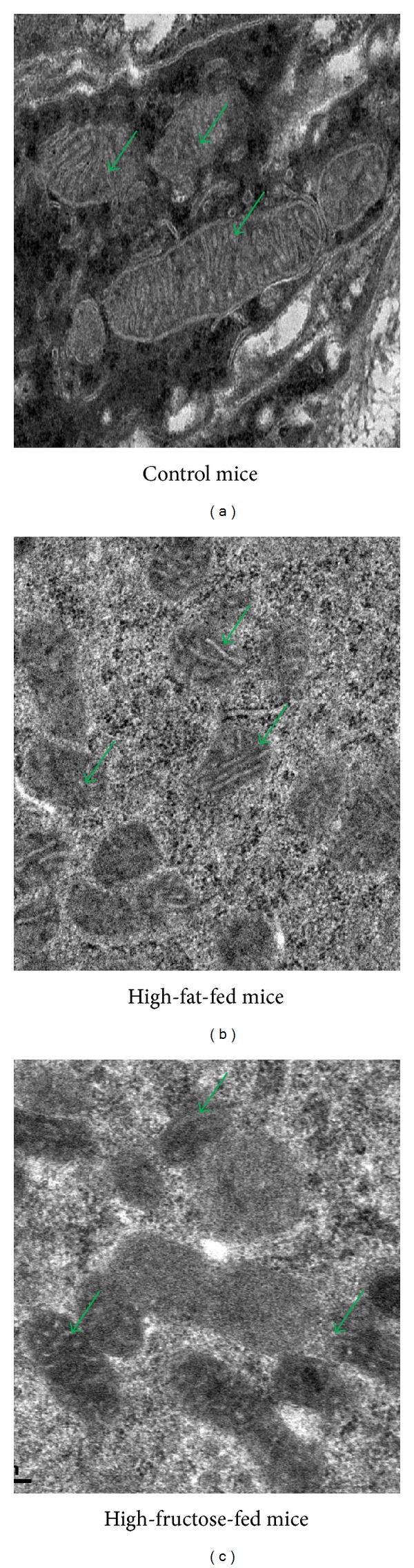
Mitochondrial ultrastructural changes in bronchial epithelia of high-fat- or high-fructose-fed mice. TEM images of airway epithelia of normal murine lung (a) show normal mitochondria (green arrows) with well-developed cristae and dense matrix compared to airway epithelia of high-fat- or high-fructose-fed mice which show the reduced number or loss of cristae (green arrows). Images are at 10500X magnification.

The role of mitochondria in asthma pathogenesis has been recently demonstrated by various studies [26, 46–49]. Mitochondrial haplogroups are associated with serum IgE levels [46], and various mitochondrial DNA mutations have been found in asthmatic patients [47, 48]. These indicate the possible causative effect of mitochondria in asthma pathogenesis. Further, we and other groups have demonstrated the mitochondrial dysfunction in asthmatic airway epithelia [26, 49]. This confirms recent hypothesis that suggested the central role of airway epithelia in asthma pathogenesis [50, 51]. Very recently, we have demonstrated that 15-LOX and its metabolites cause mitochondrial dysfunction in airway epithelia and drive severe asthmatic conditions in mice [28, 29]. This is interesting as 15-LOX and its metabolites have been shown to be crucial in metabolic syndrome [52–54]. Indeed, high-fat-fed mice showed an increase in the expression of 15-LOX which is a nonheme iron dioxygenase that catalyzes the hydroperoxidation of polyunsaturated fatty acids [52–54]. In addition, adipocyte specific deletion of 15-LOX corrected the pathological and molecular features of metabolic syndrome [53]. So we wanted to test the mitochondrial ultrastructural changes in airway epithelia in high-fat- or high-fructose-fed mice. To determine this, naïve C57BL/C mice have been given high-fat or high-fructose diet ad libitum for 20 weeks, and the harvested lungs were processed as described in Section 2. While there was no significant difference between normal- and high-fructose diet given mice in body weight, high-fat given mice had shown the doubling of body weight; however both high-fat and high-fructose given mice had shown the crucial features of metabolic syndrome such as significant increase of blood glucose, LDL cholesterol levels and increase in blood pressure (data not shown). Though we have demonstrated the mitochondrial dysfunction in airway epithelia in BALB/C mice [26], we have used C57BL/C mice for diet-induced obesity in this study. Also, we have not compared BALB/C with C57BL/C mice; however we have compared normal-diet given C57BL/C with high-energy diet given C57BL/C mice. As shown in Figure 5, normal control mice which were given control chow diet had shown the intact mitochondria with well-developed cristae and dense matrix in bronchial epithelia, whereas both high-fat- and high-fructose-fed mice had shown the loss of cristae and mitochondrial swelling. It is important to note that these mitochondrial ultrastructural changes have been observed even without any allergen sensitization and challenge. This indicated that this could be one of the reasons why obese people have a tendency to develop spontaneous airway hyperresponsiveness; however this needs further investigations. To support this view, these high-fat- or high-fructose-fed mice also showed spontaneous airway hyperresponsiveness, though these mice did not show any significant airway inflammation (data not shown). These indicate that the observed mitochondrial structural changes in bronchial epithelia in high-energy diet given mice could be inflammation independent; however this speculation needs additional investigations. Earlier studies also found the induction of spontaneous AHR in these models [55–57]. In summary, the mitochondrial dysfunction may be the crucial pathobiological phenomenon to explain abnormal lung function in obese asthmatics, and further it emphasized the importance of studying mitochondrial dysfunction and structural changes in lungs of obese asthmatics. 

### 3.4. Subepithelial Fibrosis

**Figure 6:**
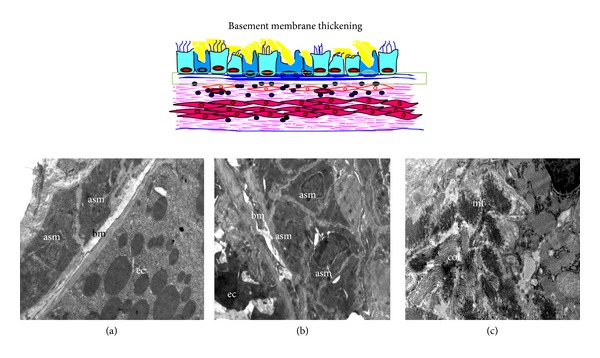
Subepithelial fibrosis. TEM of normal airway (a) shows the regular and thin basement membrane (bm), and asthmatic airways (b) and (c) show the thickened basement membrane (bm) with bundles of collagen (col) in subepithelial region and airway smooth muscle (asm) hyperplasia. ec, epithelial cell and mf, myofibroblast. Images are at 1950X magnification.

There is an interesting view that the airway inflammation followed by airway remodeling may be considered as wound healing [58]. With this view, fibrosis which occurs in the airway remodeling may be equivalent to scar in wound healing. But the major difference is the site of fibrosis. In airway remodeling, the fibrosis is not at the site of injury, that is, epithelia. Instead, the bundles of collagen (fibrosis) were found in the basement membrane and many other places such as between the smooth muscle as shown in Figure 6(b). Epithelial injury leads to release of growth factors from both structural cells and immune cells such as TGF-*β*_1_ which is a vital player of airway remodeling [2]. At early stages, eosinophil and bronchial epithelia secrete TGF-*β*_1_ which activates attenuated fibroblasts and transforms them into myofibroblasts which also start secreting TGF-*β*_1_ in later stages [59]. These secrete collagen which is deposited in the basement membrane and leads to cause thickening of basement membrane (Figure 6(b)) compared to normal mice (Figure 6(a)). In asthmatic mice, subepithelial fibrosis is composed of collagen and myofibroblast (Figure 6(c)). Structurally, myofibroblasts display a phenotype intermediate between fibroblasts and smooth muscle cells. Myofibroblast is the rich source of extracellular matrix proteins, such as collagen, fibronectin, and tenascin, each of which has been found in the subepithelium of patients with asthma [60]. Myofibroblast migration to the subepithelium may be a key factor in the development of subepithelial fibrosis. The magnitude of airway wall thickening is sufficient to contribute substantially to asthmatic AHR [61, 62]. The thickening of the basement membrane corresponds to the deposition of extracellular matrix (ECM) at subepithelial regions observed by electron microscopy [63, 64]. The true basement membrane is not grossly altered [64]. Rather, subepithelial thickening is at the lamina reticularis with deposition of the interstitial collagen I [65], III, and V [64, 66]. Though this feature is often considered as a late event, it has been reported to be found even in early asthma [67]. 

### 3.5. TEM Features of Airway Smooth Muscle Alterations (Figure 7)

**Figure 7:**
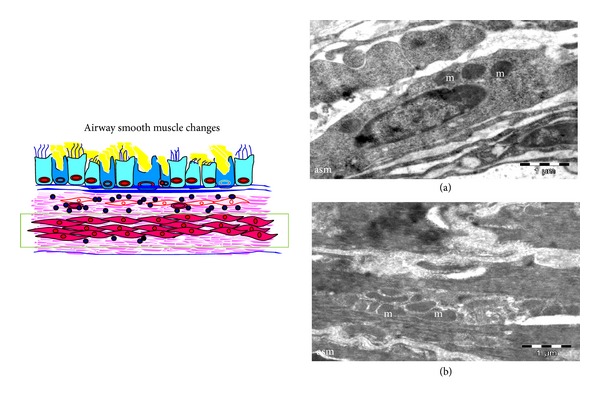
Airway Smooth muscle changes. TEM of normal airway smooth muscle (a) shows few mitochondria (m) as compared to asthmatic airway smooth muscles (asm) (b) numerous mitochondria (m). Images are at 1950X magnification.

The most prominent pathological change of airway smooth muscle in asthmatics is an increase in smooth muscle mass. Hypertrophy and hyperplasia are possible mechanisms for this increase in mass. Airway smooth muscle proliferation, as evidenced by proliferating cell nuclear antigen staining, has been found in rats and mice after repeated allergen challenge [68, 69]. Bronchial biopsies from patients with asthma reveal an increase in cell number, but not cell size [70], whereas others suggest that increased cell size is the probable mechanism. Interestingly, the site of hypertrophy varies in patients, proximal airway in one group and entire airway including bronchiole in another group. The reasons for this difference are not clear [71]. The interaction between the ECM and the airway smooth muscle cell can regulate both ECM composition and airway smooth muscle cell function. In asthmatic mice there was an increase in the size of the airway smooth muscles with an increase in the number of mitochondria (Figure 7(b)) compared to few mitochondria in normal mice (Figure 7(a)). Cell migration may also partly responsible for increase in smooth muscle mass since migration of smooth muscle cells toward the lumen of the airway underlies the appearance of myofibroblasts in the subepithelium of the asthmatic airway. In support of this hypothesis, segmental challenge of asthmatic airways increases the number of myofibroblasts in the airway subepithelium [72]. 

## 4. Conclusion and Future Perspectives

In summary, this study reveals the interesting and crucial ultrastructural changes of asthmatic airway in relation to airway remodeling such as difference in activity of eosinophils between acute and chronic models, platelet sequestration, mucus secretion from ciliated cells, widening of intermembranous space between adjacent bronchial epithelia, increased numbers of mitochondria in airway smooth muscle, and mitochondrial ultrastructural changes in bronchial epithelia. Interestingly, similar ultrastructural changes in mitochondria were also observed in lungs of mice fed with high-fat- or high-fructose diet even without allergen exposure. It is to be noted that we have not performed any asthma-related experiments in high-fat or high-fructose mice as we have not found any significant airway inflammation or goblet cell metaplasia. In light of this view, further studies are required to find whether airway epithelia of high-fat- or high-fructose-fed mice have any metabolic abnormalities such as mitochondrial dysfunction, insulin levels in these murine airways, and ultrastructural changes in high-fat and high-fructose-fed mice with allergen exposure. Nevertheless, transmission electron microscopy is a useful tool in understanding obese asthma and should be used more frequently to understand the link between asthma and metabolic syndrome.
